# Prevalence of Nasal Colonization by Methicillin-Resistant *Staphylococcus aureus* in Persons Using a Homeless Shelter in Kansas City

**DOI:** 10.3389/fpubh.2016.00234

**Published:** 2016-10-25

**Authors:** Megan Ottomeyer, Charles D. Graham, Avery D. Legg, Elizabeth S. Cooper, Chad D. Law, Mariam Molani, Karine Matevossian, Jerry Marlin, Charlott Williams, Ramon Newman, Jason A. Wasserman, Larry W. Segars, Tracey A. H. Taylor

**Affiliations:** ^1^Kansas City University of Medicine and Biosciences, Kansas City, MO, USA; ^2^Department of Internal Medicine, University of Nevada School of Medicine, Reno, NV, USA; ^3^Department of Obstetrics and Gynecology, University of Missouri-Kansas City, Kansas City, MO, USA; ^4^Children’s Mercy Hospital, Kansas City, MO, USA; ^5^Department of Biomedical Sciences, Oakland University William Beaumont School of Medicine, Rochester, MI, USA

**Keywords:** MRSA, homeless, community-acquired, *Staphylococcus aureus*, colonization

## Abstract

Nasal colonization of methicillin-resistant *Staphylococcus aureus* (MRSA) plays an important role in the epidemiology and pathogenesis of disease. Situations of close-quarter contact in groups are generally regarded as a risk factor for community-acquired MRSA strains due to transmission *via* fomites and person-to-person contact. With these criteria for risk, homeless individuals using shelter facilities, including showers and toilets, should be considered high risk for colonization and infection. The aim of this study was to determine the prevalence of nasal colonization of MRSA in a homeless population compared to established rates of colonization within the public and a control group of subjects from a neighboring medical school campus, and to analyze phylogenetic diversity among the MRSA strains. Nasal samples were taken from the study population of 332 adult participants and analyzed. In addition, participants were surveyed about various lifestyle factors in order to elucidate potential patterns of behavior associated with MRSA colonization. Homeless and control groups both had higher prevalence of MRSA (9.8 and 10.6%, respectively), when compared to the general population reported by previous studies (1.8%). However, the control group had a similar MRSA rate compared to health-care workers (4.6%), while the homeless population had an increased prevalence. Risk factors identified in this study included male gender, age over 50 years, and use of antibiotics within the past 3 months. Phylogenetic relationships between nine of the positive samples from the homeless population were analyzed, showing eight of the nine samples had a high degree of relatedness between the *spaA* genes of the MRSA strains. This indicates that the same MRSA strain might be transmitted from person-to-person among homeless population. These findings increase our understanding of key differences in MRSA characteristics within homeless populations, as well as risks for MRSA associated with being homeless, such as age and gender, which may then be a useful tool in guiding more effective prevention, treatment, and health care for homeless individuals.

## Introduction

Nasal colonization of methicillin-resistant *Staphylococcus aureus* (MRSA) plays an important role in the epidemiology and pathogenesis of disease (1). MRSA is a group of virulent strains of *S. aureus* that are resistant to treatment with beta-lactam antibiotic medicines. These strains were first reported in the United Kingdom in 1961 and appeared in the United States in 1981 (2, 3). It is estimated that 53 million people worldwide and 2.5 million Americans are carriers of MRSA (3), meaning MRSA colonizes the natural microbiota of these individuals. MRSA colonization is considered a risk for MRSA infection (4), since carrying the bacteria increases risk of infection as well as subsequent symptoms of infection, particularly if predisposing events such as an open wound or immunosuppression exist. While the clinical presentation of MRSA is identical to that of methicillin-sensitive *S. aureus*, drug resistance may present additional challenges in treatment and management (2, 3).

Methicillin-resistant *Staphylococcus aureus* is typically transmitted by skin-to-skin contact. Thus, data suggest that close-quarter environments such as prisons and schools present significant risks for transmission (4–11). Resource-poor environments, where access to healthcare is sub-optimal, also facilitate high rates of transmission (12). Homeless individuals who utilize the services of homeless shelters would therefore predictably be at higher risk for transmission, colonization, and infection due to their close proximity to each other, compounded by limited health-care access (6). Additionally, the first- and second-line treatments of MRSA infection, vancomycin and linezolid, have prohibitively high costs and might therefore be unavailable to this population (7–10). This is expected to result in a greater proportion of chronic MRSA infection and colonization among the homeless population.

Rates of homelessness in the United States historically have remained relatively low, except for temporary increases during economic recessions in the late 1800s and during the great depression (13). However, beginning in the 1970s, economic recession combined with wages that stagnated against inflation has engendered a larger and rather persistent homeless population in the United States (13, 14). The American societal response to rising rates of homelessness from the 1980s and into the early 2000s centered on providing shelter services and other programs that involved communal living (13, 14). Despite a more recent focus on “rapid rehousing,” these types of services largely continue to serve as a gateway to permanent housing. The health risks associated with these environments therefore remain of great interest (15).

Overall medical illness rates among homeless populations tend to be higher than the rates among housed individuals (15–19). Taking into account all possible causes, the mortality risk in homeless subjects was found to be 4.4 times that of a non-homeless cohort in one retrospective study (18). Importantly, incidences of diabetes mellitus, HIV, and tuberculosis have shown higher rates among homeless individuals (15–17). These illnesses likely play a role in development of MRSA infection by means of immunosuppression.

While it has been shown that the homeless population have higher rates of various diseases, very few studies have investigated MRSA colonization and infection rates among this population. In addition, the majority of published studies have been limited to a hospital setting (predominantly emergency department admissions) (10), making it difficult to understand the role homelessness itself plays in MRSA prevalence and how health-care providers can incorporate a patient’s homeless status and particular living situation (e.g., on the street versus in a shelter) as a factor in guiding diagnosis and treatment. This study attempts to fill these gaps, by exploring the association of homelessness to MRSA colonization status in non-hospitalized individuals, and to determine other possible risk factors for MRSA colonization in a homeless population. Additionally, the genetic similarity between MRSA strains isolated from a sample of study participants was examined by phylogenetic analysis to determine the likelihood of person-to-person transmission of one strain versus colonization of various strains from different environments. The findings from this study will provide further evidence of the role of group living situations on colonization by MRSA.

## Materials and Methods

The major aim of this study was to explore the association of homelessness to MRSA colonization status in non-hospitalized individuals. Using collected survey response results, we further aimed to determine other possible risk factors for MRSA colonization in a homeless population in the Midwestern United States. Lastly, our third aim was to utilize phylogenetic analysis to determine the genetic similarity between a subset of MRSA strains isolated from study participants.

### Subject Recruitment: Homeless Shelter

Data were gathered over 10 visits (July 2012–June 2014) to a homeless aid facility in the Midwestern United States that provides food, shelter, clothing, and employment training services to several hundred homeless and economically disadvantaged people daily and up to approximately 10,000 people per year. The study was advertised with informational flyers on the day prior and day of collection. Subjects self-referred to the sample collection location for a free health screening and sample collection (nasal swab) following written informed consent. After sample collection, a short survey was administered to collect demographic and risk factor data including daily habits, antibiotic use, and incarceration (details of the complete survey used in this study is provided in Figure S1 in Supplementary Material). All survey and sample collection methods, and subject written informed consent, were approved by Kansas City University of Medicine and Biosciences’ local Internal Review Board (IRB study number 330726).

### Subject Recruitment: Control Study

Data were gathered over two separate collection events at a medical school in the Midwestern United States. The study was advertised 2 days prior and on the day of the study with informational fliers and an online posting. Subjects, which included students, faculty, staff of the school, volunteered for sample collection following written informed consent and completed the identical IRB-approved survey used in the experimental study.

### Sample Collection and Culture

Nasal samples were collected from homeless and control adult subjects using Rayon tipped Remel^®^ Bacti-swabs with Stuart’s media ampules. After acquiring written informed consent, the swabs were inserted into the nares to a depth of approximately 1/4″ and swirled using a twisting and stirring motion. The Stuart’s media ampules were then broken and the swabs stored in a cooler with cold packs (approximately 4°C) until they could be plated as described in section below (Specimen Analysis), no more than 3 h later.

### Surface Culture

Samples from 10 surfaces were collected using BD Cultureswab EZ^®^ polyurethane foam tipped swabs. Samples were collected from surfaces of frequent hand contact including a stair rail, chair handles, a men’s room flush button, a refrigerator handle, a telephone, a television remote control, a computer keyboard and mouse, a door handle, and a light switch. Ten additional control samples were collected from analogous surfaces using the same technique at the Kansas City University of Medicine and Biosciences campus for a comparative sampling. Swabs were then plated as described in section below (Specimen Analysis).

### Specimen Analysis

All nasal collection swabs and surface swabs were plated directly from swabs first onto HardyChrom™ MRSA plates, then onto BBL^®^ tryptic soy agar (TSA) plates. Plates were placed in a 37°C incubator for 24 h. Samples demonstrating growth on the HardyChrom™ MRSA plates were subjected to catalase testing using 3% hydrogen peroxide, coagulase testing with Remel^®^ coagulase plasma (rabbit), and visualized microscopically under Gram stain to confirm identity as *S. aureus* (Gram-positive cocci in clusters). Control strains used included ATCC 25923 methicillin-sensitive *S. aureus* and ATCC 43300 MRSA for positive Staphylococcal controls and ATCC 25175 *Streptococcus mutans* as a negative control for catalase and coagulase tests. Additionally, ATCC 25923 methicillin-sensitive *S. aureus* and ATCC 43300 MRSA were used as negative and positive controls, respectively, on HardyChrom™ MRSA plates.

### Phylogenetic Analysis

Nine of the samples positive for MRSA from the initial screening were grown on TSA plates at 37°C for 48 h. Following isolation of chromosomal DNA using standard laboratory procedures, the DNA samples were then subjected to PCR for the *spaA* gene with the following primers (20).

*spaA* F: 5′-AGACGATCCTTCGGTGAGC
*spaA* R: 5′-GCTTTTGCAATGTCATTTACTG

The PCR products were then excised and extracted from agarose gels and sent for sequencing (Genewiz). Sequences were analyzed using the Phylogeny-fr database (21, 22) to determine phylogenic relationships between the MRSA strains in the samples.

### Statistical Analysis

Data from the cultures and surveys were analyzed using SPSS (Version 22.0, SPSS Inc., Chicago, IL, USA) as well as Graph Pad Prism 6. Differences in relative risk between populations were analyzed using a chi-squared test.

## Results

### Prevalence of MRSA

Using a total of 285 samples from the homeless population and the 47 samples from the control population, it was determined by chi-squared analysis that both the homeless population (*p* < 0.001) and the control group (*p* < 0.01) had an increased prevalence of MRSA colonization compared to the general population (Table 1) as reported by Dulon et al. in a 2014 study (23). However, since the control study was at a medical school campus and included both medical students and practicing medical faculty, it is reasonable to believe that some of the subjects have had equivalent exposure to MRSA as United States health-care workers. When the prevalence of MRSA colonization of the control group was compared to that of the reported prevalence in health-care workers by chi-squared analysis (23), there was not a significant difference in prevalence (*p* = 0.0639).

**Table 1:** **Prevalence of methicillin-resistant *Staphylococcus aureus* (MRSA) colonization of control and homeless populations compared to prevalence in healthcare workers and the general population**.

Population	MRSA+	MRSA−	Prevalence	Relative risk
Homeless	28	257	0.09825	5.458
General population	–	–	0.018^a^	

Control	5	42	0.10638	2.3126
Health-care workers	–	–	0.046^a^	

*^a^Values from Ref. (23)*.

Prevalence significantly increased in homeless population compared to general population (*p* < 0.00001). Control population also was significantly increased compared to general population (*p* = 0.0015) but not when compared to health-care workers (*p* = 0.0639).

### Analysis of Surfaces for MRSA

Samples from 10 surfaces were collected from the participating homeless shelter, as well as samples from 10 analogous surfaces at the location of the control study. Of the samples taken from both locations, there were no methicillin-resistant colonies of *S. aureus* isolated, suggesting that fomites did not serve as a significant source of MRSA transmission in either study group (Table 2).

**Table 2:** **Overall growth and growth of methicillin-resistant *Staphylococcus aureus* (MRSA) from samples collected from surfaces**.

Surface	Shelter			Control		
	Growth on TSA^a^	HardyChrom™ colonies	MRSA+^b^	Growth on TSA	HardyChrom™ colonies	MRSA+^b^
Stair rail	+++	4	0	++	0	0

Light switch	+	0	0	++	0	0

Refrigerator handle	++++	0	0	+++	3	0

Telephone	++	2	0	++	0	0

TV remote	++	2	0	+	1	0

Door handle	++	0	0	+	0	0

Chair handle	++	0	0	+	0	0

Toilet flush button	++	0	0	+++	4	0

Keyboard	+++	3	0	++	0	0

Computer mouse	+	0	0	++	0	0

*^a^TSA, tryptic soy agar media*.

^b^Colonies confirmed to be MRSA via further testing as described in Section “Materials and Methods.”

### Subject Demographics and MRSA Colonization

Identical surveys were administered to all study participants (Figure S1 in Supplementary Material). Surveys included questions regarding basic information such as age and gender, as well as ratings of physical and mental health, daily activities, health history, and social history including history of incarceration. Overall survey responses were used to determine potential risk factors for MRSA colonization (Table 3).

**Table 3:** **Overall survey responses, relative risk, and attributable risk associated with selected surveyed lifestyle factors**.

Risk factor	MRSA+ (%)^a^	MRSA−	Total	Relative risk	Attributable risk
Homeless	28 (9.8%)	257	285	0.923509	−0.00814
Control	5 (10.6%)	42	47		

Homeless over 50 years old	12 (11.8%)	90	102	1.345588	0.030215
Homeless under 50 years old	16 (8.7%)	167	183		

Homeless male over 50 years old	8 (10.7%)	67	75	1.091282	0.008922
Homeless male under 50 years old	13 (9.8%)	120	133		

Homeless male over 50 years old	8 (10.7%)	67	75	0.773333	−0.03126
Homeless female over 50 years old	4 (13.8%)	25	29		

Homeless over 50 years old	12 (11.8%)	90	102	2.352941	0.067647
Control over 50 years old	1 (5%)	19	20		

Male	25 (10.7%)	208	233	1.32779	0.026488
Female	8 (8.1%)	91	99		

Homeless male	21 (10.1%)	187	208	1.110577	0.010052
Homeless female	7 (9.1%)	70	77		

Homeless male	21 (10.1%)	187	208	0.63101	−0.05904
Control male	4 (16%)	21	25		

Homeless with antibiotic use history (past 3 months)	8 (10.5%)	68	76	1.089474	0.008645
Homeless without antibiotic use history (past 3 months)	20 (9.7%)	187	207		

Homeless with antibiotic use history (past 3 months)	8 (10.5%)	68	76	1.157895	0.014354
Control with antibiotic use history (past 3 months)	1 (9.1%)	10	11		

*^a^MRSA, methicillin-resistant Staphylococcus aureus*.

**Figure 1:**
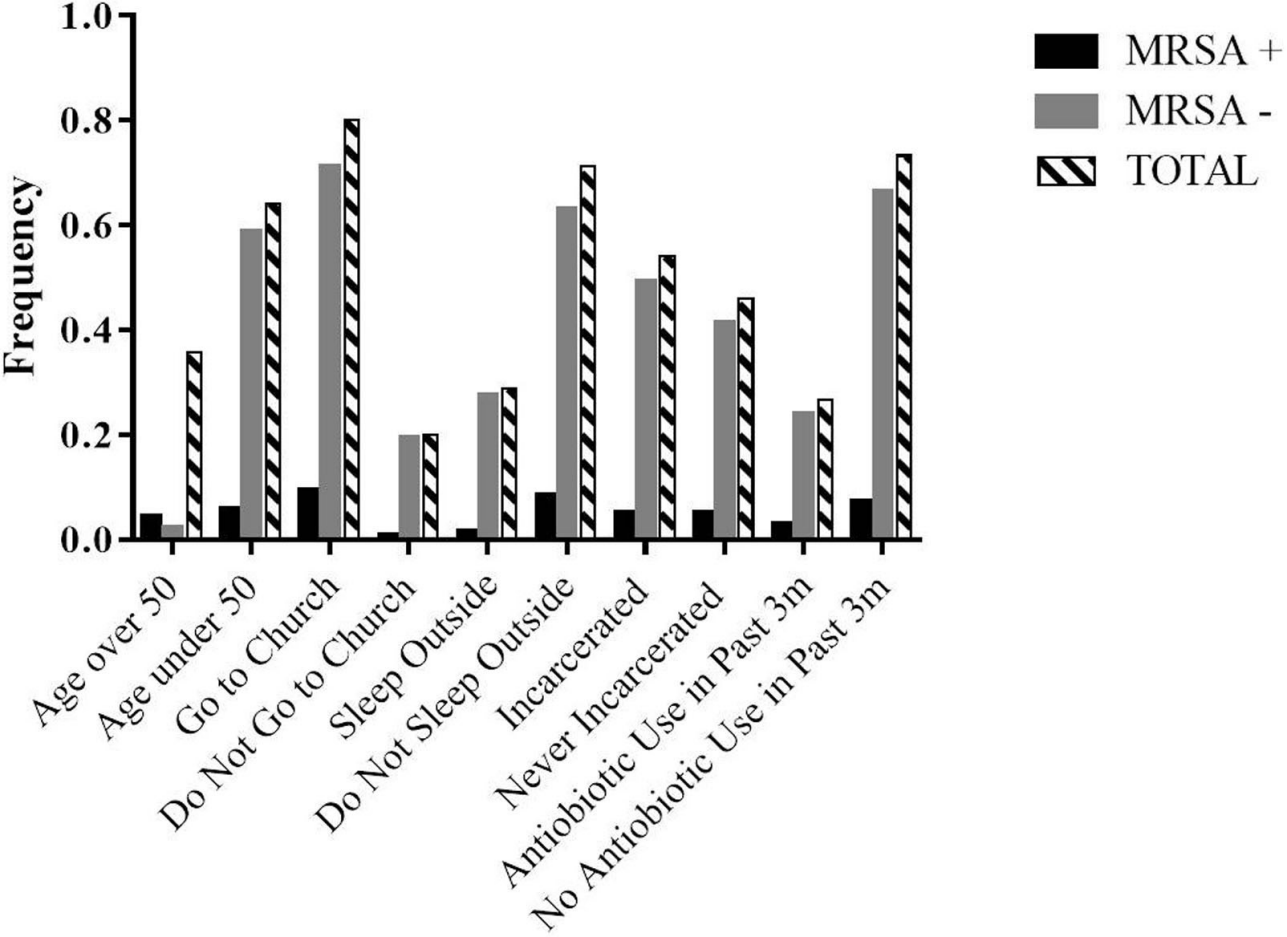
**Response frequencies for selected surveyed lifestyle factors and MRSA status in the homeless population**. Participation in activities was collected as days in an average week with greater than or equal to 1 day a week being positive for an activity and less than 1 day a week being negative for a given activity.

After a preliminary review of those responses and of the current literature, it was hypothesized that potential risk factors for MRSA colonization included age, gender, history of incarceration, and antibiotic use within the past 3 months for the homeless population (Figure 1), so these responses were investigated in more depth. Age over 50 years was found to be a risk factor for MRSA colonization in the homeless population, with a relative risk of 1.34 and an attributable risk of 3.02% (Table 3). It was also determined that both in the control and homeless populations, more males were colonized with MRSA than females, with male gender having a relative risk of 1.33 with both the control and homeless populations combined and a relative risk of 1.11 in the homeless population. Male gender also had an attributable risk of 2.6 and 1.0% in the combined populations and homeless population, respectively (Table 3).

Surprisingly, history of incarceration in the homeless population did not prove to be a strong risk factor for MRSA colonization (Table 4), despite the crowding conditions associated with incarceration. Antibiotic use within the last 3 months was more common in the homeless population and relative risk for MRSA positivity in the homeless population was 1.09 (Table 3). Additionally, the relative risk of MRSA positivity between homeless and control subjects with history of recent antibiotic use was 1.16 with an attributable risk of 1.4% (Table 3).

**Table 4:** **Comparison of selected surveyed lifestyle factors and demographics in subjects colonized with related MRSA strain (*n* = 8) to unrelated MRSA strain (*n* = 1)**.

Lifestyle factor	Related MRSA strain	Unrelated MRSA strain
Average shelter use per week (days)	5.5	7

Previous incarceration (%)	87.50	0

Greater than age 50 (%)	87.50	0

Seen doctor in past year (%)	75	100

Seen dentist in past year (%)	25	100

Skin infection in past 3 months (%)	12.50	100

Antibiotic use in past 3 months (%)	25	0

**Figure 2:**
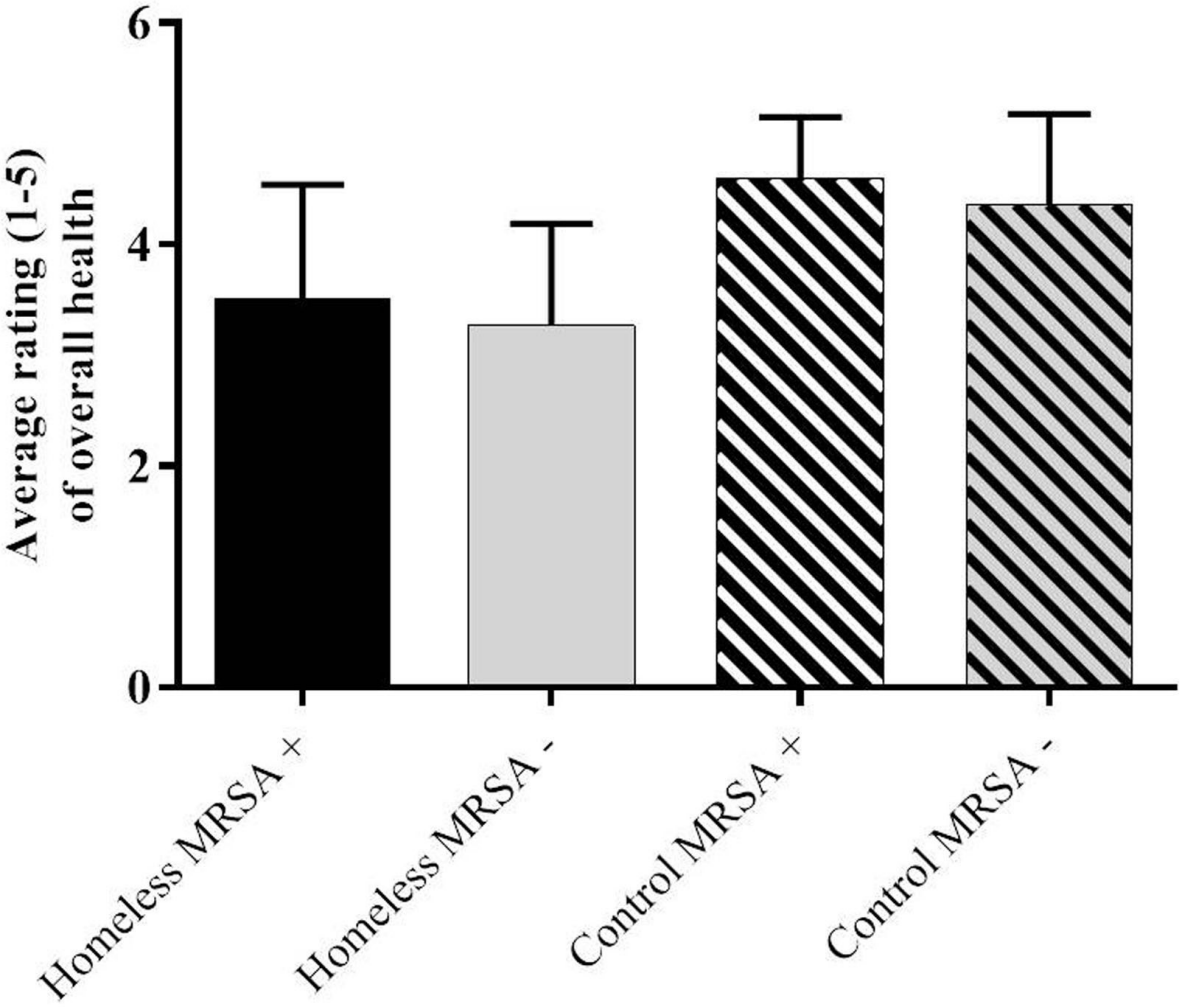
**Average rating of self-perceived overall health on a scale of 1–5 with 1 being very poor and 5 being very good**.

Interestingly, despite recent antibiotic use being more common overall in the homeless population, the average rating of overall self-perceived health did not differ significantly among the homeless and control populations (Figure 2), although the control population perceived their health to be slightly higher than the homeless population. This may be due to different expectations of health against which each participant would benchmark the response options in the survey.

### Phylogenetic Analysis

**Figure 3:**
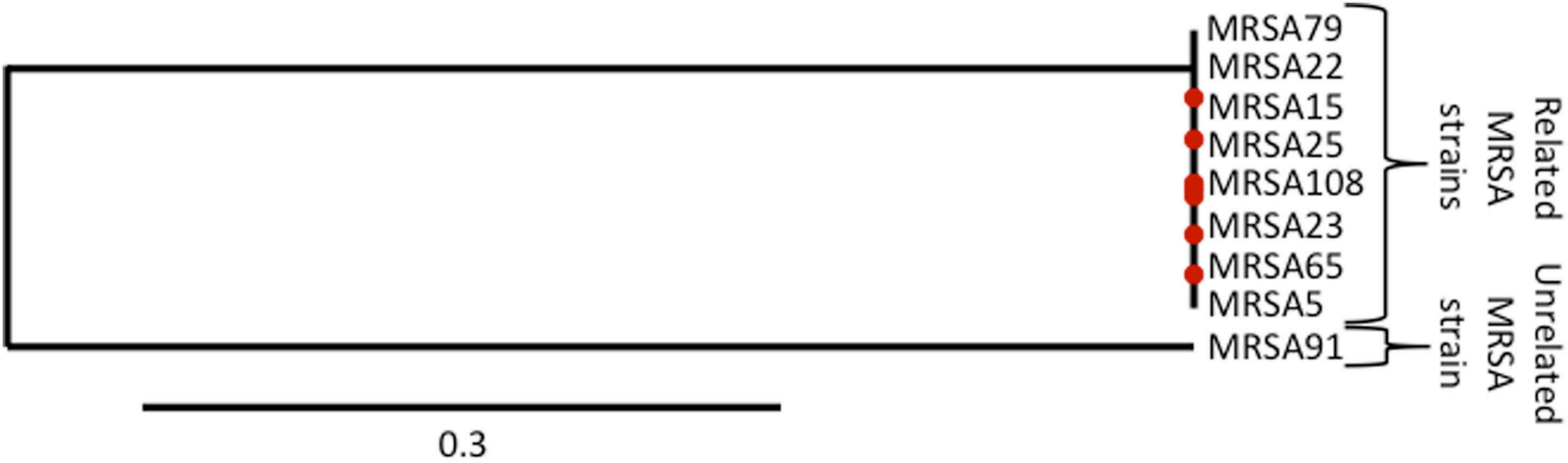
**Phylogeny of selected MRSA samples from homeless population by *spaA* gene**.

The *spaA* gene sequences of nine MRSA positive samples from the homeless population were used to determine relatedness of MRSA strain between the samples (Figure 3). Of the nine samples, eight were closely related (samples 5, 15, 22, 23, 25, 65, 79, and 108) and one was unrelated (sample 91). Differences in lifestyle factors as well as demographic information from these subjects were compared (Table 4).

Key differences between subjects with the related strain and the subject with the unrelated strain included history of incarceration, age, history of recent skin infection, and seeing a dentist within the past year. These factors may signal either shared experiences or shared attendance at particular services. However, a single point of shared colonization origin could not be determined from the scope of this study, mostly due to inability to follow-up with specific study participants and anonymity of subjects.

## Discussion

The goal of this study was to investigate the prevalence of MRSA colonization in non-hospitalized homeless individuals, as well as to determine other risk factors for colonization. We further characterized the genetic similarity between MRSA strains isolated from study participants using phylogenetic analysis. Given the crowded conditions of shelters and lifestyle factors associated with homelessness, homeless individuals appear to be at a higher risk for MRSA colonization than the general population (Table 1). This supports the retrospective findings of Farr et al. and Young et al. who reported homelessness as a risk factor for MRSA colonization in hospitalized individuals (24, 25). In addition, MRSA colonization among homeless individuals is likely due to community-acquired colonization by person-to-person transmission or transmission *via* shared fomites, as the strains are closely related phylogenetically (Figure 3); however, our survey of common surfaces (Table 2) did not reveal a source within the shelter itself, which suggests an alternative origin of MRSA-colonized shared fomites or that person-to-person transmission of MRSA is more likely.

The identification of male gender, age over 50 years, and recent antibiotic use as risk factors for the homeless population (Figure 1; Tables 3 and 4) allows for a clearer identification of those homeless people that may be at an increased risk of MRSA infection and could thus allow for more thorough follow-up with those individuals to either prevent or treat a MRSA infection. For example, if a male patient over 50 years of age known to be homeless were to be placed on antibiotics by a health-care provider, that provider would be able to consider the patient’s risk factors as increased for MRSA colonization and infection and suggest a follow-up appointment to assess for MRSA infection or educate the patient of certain signs or symptoms that may suggest a MRSA infection in the future.

Given that our survey results demonstrated that recent antibiotic use (in the previous 3 months) was more common overall in the homeless population when compared to our control population, we were surprised to find that the average rating of overall self-perceived health did not differ significantly (Figure 2). The control population perceived their health to be slightly higher than the homeless population. We presume that expectations of health are higher among the control population when compared to the homeless population (26).

Further work is needed to determine if risk factors for MRSA colonization of the homeless differ geographically, seasonally, or in relationship to other environmental factors. This may help health-care workers consider MRSA infection as a differential diagnosis when treating homeless patients or patients with similar risk factors for MRSA colonization. A greater sample size also should be collected that would allow the sample to be partitioned in additional ways that might highlight other factors that promote risk of MRSA colonization among the homeless population. Additionally, because our sample size was limited, our data were compared to that of the pre-existing literature (which varies greatly), whereas a comparison to a control group with more similar demographics would be ideal. Finally, future research should aim to characterize on the molecular level whether the MRSA found in this population is of community-acquired or health-care-associated origin.

## Conclusion

For its part, this study is unique in determining risk factors for MRSA colonization in a non-hospitalized, medically underserved, and poorly represented population in previous literature in real time, rather than through retrospective studies using only hospital records. Additionally, it provides guidance for future studies to explore specific risk factors for MRSA colonization in more depth, which could be helpful in better understanding and treating homeless patients.

## Ethics Approval and Consent to Participate

This study was carried out in accordance with the recommendations of Kansas City University of Medicine and Biosciences’ local Internal Review Board (IRB study number 330726) with written informed consent from all subjects. All subjects gave written informed consent in accordance with the Declaration of Helsinki.

## Author Contributions

Although we acknowledge that our author list is lengthy, all authors fulfill the International Committee of Medical Journal Editors criteria for authorship. MO was involved with analysis of the experiments as well as writing and editing of the manuscript. CG was heavily involved with conception, development, and execution, as well as initial writing of the manuscript. AL, EC, CL, MM, and KM were involved with execution and analysis of the experiments as well as editing of the manuscript. JM was involved with execution of the experiments and editing of the manuscript. CW and RN were heavily involved with execution, and their presence and training of the medical students was required in order for us to collect data. JW and LS are statistics experts whose expertise was required in order to analyze the collected data. TT is the principle investigator and was involved with conception, development, and analysis of the experiments, as well as writing and editing of the manuscript. All the authors read and approved the final manuscript.

## Conflict of Interest Statement

The authors declare that the research was conducted in the absence of any commercial or financial relationships that could be construed as a potential conflict of interest.

## Supplementary Material

Figure S1 **Prevalence of MRSA in homeless subjects of Kansas City questionnaire**. This is the complete KCU IRB-approved survey used in this study for both the study group and the control group.

## Abbreviations

HIV, human immunodeficiency virus; IRB, Institutional Review Board; KCU, Kansas City University of Medicine and Biosciences; MRSA, methicillin-resistant *Staphylococcus aureus*; TSA, tryptic soy agar.
