# Supplementary material for: Prevalence of Nasal Colonization by Methicillin-Resistant Staphylococcus aureus in Persons Using a Homeless Shelter in Kansas City
Source: Front Public Health. 2016 Oct 25;4:234. doi: 10.3389/fpubh.2016.00234 (PMC5078475; doi:10.3389/fpubh.2016.00234)
Supplement: Figure S1 — Prevalence of MRSA in homeless subjects of Kansas City questionnaire. This is the complete KCU IRB-approved survey used in this study for both the study group and the control group. [file Image_1.PDF]

Prevalence of MRSA in homeless subjects of Kansas City questionnaire

Age____ Sex_______ Occupation______________ Months Homeless_____ Swab#_____

What is your education history: (S = some, C = complete, E = currently enrolled)

Elem. school____ Middle school____ High school____ College/Trade school____ Grad school____

Would you say that you typically spend more time:

Alone With family In a small group In a large group

During a typical week, how many times do you visit/engage in the following:

Shelter____ Library____ Thrift store____ Grocery store____ Convenience store____

Public transportation____ Church____ Train/bus station____ Post office____ Sleeping outside____

Restaurant____ Public park____ Department store____ Moderate exercise____

Have you ever been incarcerated Y N When (most recent)_______ For how long (total)______

Have you ever been hospitalized Y N When (most recent)_______ For how long (total)______

Regarding your overall stress level for the last three months, how often have you felt:

Out of control?	Never	Almost never	Sometimes	Almost always	Always
Sick?	Never	Almost never	Sometimes	Almost always	Always
Overwhelmed?	Never	Almost never	Sometimes	Almost always	Always
Optimistic?	Never	Almost never	Sometimes	Almost always	Always
Things going your way?	Never	Almost never	Sometimes	Almost always	Always

How would you describe your overall health:

Very good Good Average Poor Very poor

How many times in the last year have you seen: Doctor_____ Dentist_____

In the last three months, have you: Had a skin infection______ Used antibiotics_____

Where have you received the majority of your healthcare:

Emergency room Doctors Office Free clinic Other

How easy is it for you to get dental care when you need it:

Very easy Somewhat easy Somewhat difficult Very difficult

Compared to the average person, would you say that doctors and dentists are:

More trustworthy About as trustworthy Less trustworthy
